# When the brain fades before the eye: encephalopathy as a rare presentation of direct carotid-cavernous fistula

**DOI:** 10.1055/s-0045-1809932

**Published:** 2025-07-15

**Authors:** Leonardo Furtado Freitas, Eduardo J. Labat, Robert T. Wicks, Charif Sidani, Kevin J. Abrams

**Affiliations:** 1 Baptist Health South Florida, Department of Radiology, Division of Clinical Neuroradiology, Miami FL, United States.; 2 Florida International University, Herbert Wertheim College of Medicine, Miami FL, United States.; 3 Baptist Health South Florida, Department of Cerebrovascular Neurosurgery, Miami FL, United States.

**Figure 1:**
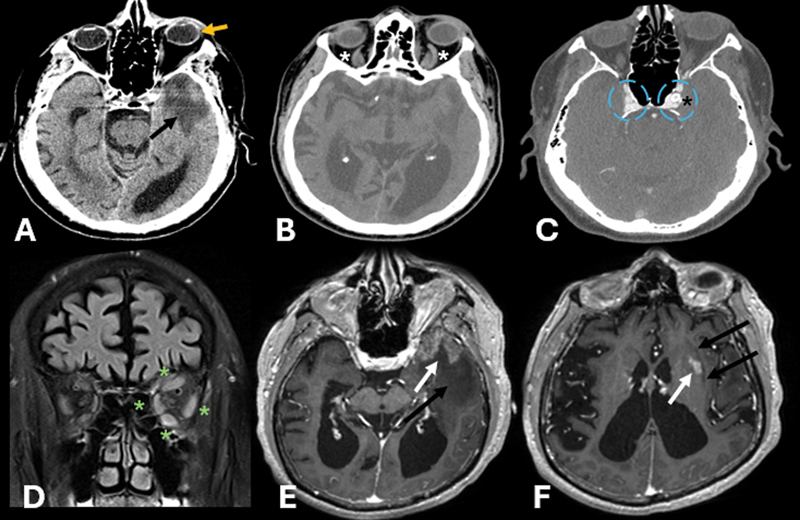
Axial non-contrast computed tomography (CT) scan ( **A, B** ) and CT angiography (CTA) ( **C** ). Coronal orbit magnetic resonance imaging (MRI) scan on fluid-attenuated inversion recovery (FLAIR)-weighted images with fat suppression ( **D** ) and brain MRI scan on post-contrast axial T1 gradient echo-weighted images ( **E,F** ). Left temporal hypoattenuation with proptosis (black and orange arrows), dilation of the superior ophthalmic veins (white asterisks), and a stent in the cavernous segment of the left internal carotid artery (black asterisk) with cavernous sinuses engorgement (dashed blue circles). MRI scan showing prominent left temporal vasogenic edema extending to the subinsular region and putamen (black arrows), edema in the left extraocular muscles (green asterisks). There is also thick cortical enhancement in the left temporal pole and nodular enhancement in the ipsilateral putamen (white arrows).

**Figure 2:**
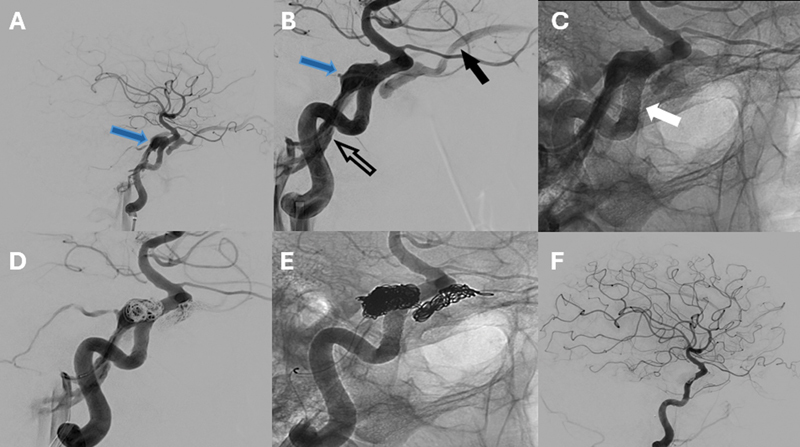
Cerebral angiogram. Left internal carotid artery, lateral projection ( **A** ), revealing direct carotid cavernous fistula with contrast opacification of the cavernous sinus (blue arrow). Left internal carotid artery injection, magnified lateral projection ( **B** ) – the solid arrow denotes dilated superior ophthalmic vein. The hollow and blue arrows show dilated inferior petrosal sinus and cavernous sinus, respectively. Left Internal carotid artery injection, unsubtracted lateral projection ( **C** ), which enables the visualization of internal carotid artery cavernous segment stent (white arrow). Left internal carotid artery injection, lateral projection ( **D** ). Intraprocedural coiling of carotid cavernous fistula via the transvenous approach through the inferior petrosal sinus. Left internal carotid artery injection, unsubtracted lateral projection ( **E** ). Posttransvenous coil embolization of carotid cavernous fistula. Left internal carotid artery injection, ateral projections ( **F** ), revealing complete coil embolization of the carotid cavernous fistula.

We herein report a rare case of a 76-year-old male patient with progressive cognitive decline, acute encephalopathy, and visual symptoms 6 months after cavernous internal carotid artery (ICA) stenting for intracranial stenosis. The initial workup was negative. Neuroimaging ( Figure 1 ) showed left temporal and orbital edema with cavernous sinuses abnormalities. The ophthalmologic exam revealed palsy of the left cranial nerve VI and elevated intraocular pressure. Cerebral angiography ( Figure 2 ) confirmed a direct left-sided, type-A carotid-cavernous fistula (CCF) with bilateral venous drainage. Endovascular embolization resulted in clinical improvement. This case highlights an uncommon presentation of direct CCF with predominant encephalopathy 1 2 and emphasizes the importance of considering vascular causes in atypical cognitive decline following ICA stenting.
